# Nonhormonal Hot Flash Management for Breast Cancer Survivors: A Systematic Review and Network Meta-Analysis

**DOI:** 10.1155/2020/4243175

**Published:** 2020-04-28

**Authors:** Jian Liu, Guangning Nie, Yang Li, Zehuai Wen, Liming Lu, Li Xie, Dongdong Cao, Yafang Lai, Hongyan Yang

**Affiliations:** ^1^Department of Gynecology, The Second Affiliated Hospital of Guangzhou University of Chinese Medicine, Dade Road, Yuexiu District, Guangzhou 510120, Guangdong, China; ^2^Key Unit of Methodology in Clinical Research, The Second Affiliated Hospital of Guangzhou University of Chinese Medicine, Guangzhou, Guangdong, China; ^3^Clinical Research Center, South China Research Center for Acupuncture and Moxibustion, Medical College of Acu-Moxi and Rehabilitation, Guangzhou University of Chinese Medicine, Guangzhou, Guangdong, China; ^4^The Second Clinical College of Guangzhou University of Chinese Medicine, Guangzhou, Guangdong, China; ^5^Department of Chinese Medicine, Affiliated Hospital of Guilin Medical University, Guilin, Guangxi, China; ^6^Department of Gynecology, Foshan Hospital of Traditional Chinese Medicine, Guangzhou, Guangdong, China; ^7^Guangdong Provincial Key Laboratory of Clinical Research on Traditional Chinese Medicine Syndrome, The Second Affiliated Hospital of Guangzhou University of Chinese Medicine, Guangzhou, Guangdong, China

## Abstract

**Materials and Methods:**

We conducted a systematic literature search in PubMed, Cochrane Central Register of Controlled Trials, Embase, Chinese Biomedicine Database (CBM), China National Knowledge Infrastructure (CNKI), Wan Fang, and VIP up to May 2018. Randomized controlled trials (RCTs) reporting nonhormonal hot flash treatments for breast cancer survivors were included. Primary outcome measurements were hot flash frequency and hot flash score of posttreatment. The methodological quality of each study was assessed with Cochrane's risk of bias tool.

**Results:**

16 RCTs involving 2,349 participants were included. The nonhormonal therapies used in the included studies were classified as follows: lifestyle changes, mind-body techniques, dietary/supplements, SSRIs/SNRIs, other medications, and other therapies. Pairwise meta-analysis showed that the general effect of nonhormonal management was statistically more effective than no treatment/placebo/sham in reducing hot flash frequency (SMD = −0.60, 95% CI [−1.13, −0.06]; *P*=0.03)) and hot flash score (SMD = −0.38, 95% CI [−0.68, −0.08]). For hot flash frequency, results from the NMA showed that there was no statistically significant difference between any two of the nonhormonal treatments. Another NMA result indicated that acupuncture (other therapies) was 16.05 points more effective in reducing hot flash scores than no treatment/waitlist (SMD = −16.05, 95% CI [−30.2, −1.99]). These results were statistically significant. Acupuncture was also ranked the optimal nonhormonal therapy for both hot flash frequency and hot flash score. The safety analysis showed that there were few related adverse events during acupuncture and that drug related adverse reactions could have also occurred in studies using drug interventions

**Conclusions:**

This network meta-analysis comparing nonhormonal treatments suggested that acupuncture might be more effective in improving hot flashes for breast cancer survivors. A pronounced placebo response was found during hot flash treatments. The evidence of safety for nonhormonal therapies was also insufficient. Therefore, at present, we cannot make confirmative recommendations of nonhormonal hot flash management for breast cancer survivors. This study is registered with PROSPERO (CRD42018082008).

## 1. Introduction

Hot flashes are common among breast cancer survivors [1]. This population has more severe and persistent hot flashes due to premature menopause resulting from chemotherapy and the use of endocrine therapies such as tamoxifen and aromatase inhibitors [2, 3]. It has been demonstrated that 72% of tamoxifen recipients and 78% of chemotherapy recipients experience hot flashes [4]. Breast cancer survivors who experience hot flashes also tend to experience more fatigue, worse sleep quality, and lower quality of life [5–7].

Because of concerns that estrogen exposure increases cancer recurrence rates, menopausal hormonal therapy (HT) is not the recommended first-line hot flash treatment for breast cancer survivors [8]. This leaves fewer hot flash treatment options than women without a history of breast cancer. Hence, nonhormonal medicinal treatments (e.g., antidepressants) [9, 10], physical/behavioral treatments (e.g., yoga/exercise, cognitive behavioral therapy) [11, 12], acupuncture [13, 14], and natural health products (e.g., black cohosh, soy) [15, 16] have been used for hot flashes. Several systematic reviews have summarized studies and data comparing these interventions, both for placebos and for controls [17–20], and have provided favorable recommendations of particular interventions.

The 2015 position statement of the North American Menopause Society [21] indicated that nonhormonal management is now a realistic option for women who cannot take estrogen. In this statement, nonhormonal management of menopause-associated hot flashes is classified into the following categories: lifestyle changes, mind-body techniques, dietary management and supplements, SSRIs/SNRIs, other medications, and other therapies. For clinical decision making, all potential interactions with management treatments need to be weighed against potential effectiveness. While past reviews have considered some nonhormonal treatments in isolation [22], we performed a network meta-analysis [23, 24] to compare many alternatives in a unified analysis. This study may provide data to guide physician and patient choice of intervention by efficacy or by side-effect profile.

## 2. Methods

### 2.1. Protocol and Registration

This research followed the Preferred Reporting Items for Systematic Reviews and Meta-Analyses for Network Meta-Analysis (PRISMA-NMA) checklist [25]. The protocol was registered on the International Prospective Register of Systematic Reviews (PROSPERO; registration number: CRD42018082008).

### 2.2. Inclusion and Exclusion Criteria

We looked for the following inclusion criteria: (1) participants: women who had been diagnosed with breast cancer and who were experiencing hot flashes, without restrictions on age or cancer stage; (2) intervention: nonhormonal therapies (classified by the 2015 position statement of the North American Menopause Society [21] including lifestyle changes, mind-body techniques, dietary/supplements, SSRIs/SNRIs, other medications, and other therapies; (3) comparison: hormonal therapies, no treatment/waitlist, placebo/sham treatment; (4) outcomes: hot flash frequency or hot flash scores (frequency × mean severity or measured using scales such as the hot flash diary) of posttreatment, adverse events; (5) study design: randomized clinical trial (RCT); (6) language: English or Chinese studies.

We excluded studies based on the following criteria: (1) participants with multiple forms of cancer; (2) participants who had not received nonhormonal therapy; (3) self-control and non-RCTs; (4) preclinical studies, systematic reviews, case reports, and meta-analyses; (5) protocols, unpublished or duplicate studies.

### 2.3. Information Sources and Search Strategy

We systematically searched the following databases: PubMed, Cochrane Central Register of Controlled Trials, Embase, Chinese Biomedicine Database (CBM), China National Knowledge Infrastructure (CNKI), Wan Fang, and VIP. We identified articles published from journal inception to May 2018, with a limit to studies of RCTs and without limitations on language or publishing format. A more detailed description of the search strategies is presented in Supplementary Material 1.

### 2.4. Study Selection

Two authors of the review (YL and DDC) independently identified relevant research based on titles and abstracts. Full-text articles were then scanned by four researchers to identify eligible studies. All disagreements were resolved by consensus and adjudicated by a third reviewer (GNN), if necessary. In cases of duplicate citations, the most updated studies were selected for data extraction.

### 2.5. Data Extraction

According to a standard data collection sheet, two reviewers (LX and YFL) independently extracted data on each included article. Extracted data included year of publication, author information, patient characteristics, sample size, arms of interest, interventions, outcomes, and adverse effect measures and results. In some trials, the data of posttreatment were not given. We used the methods recommended in the Cochrane Handbook for Systematic Reviews of Interventions (version 5.1) [26] to resolve it. Information from studies identified in other reviews was subsequently cross-checked. Inconsistencies and uncertainties were resolved by consultation with a third reviewer (GNN).

### 2.6. Quality Assessment

Two of the authors (JL, GNN) independently used the Cochrane risk of bias tool, as described in the Cochrane Handbook for Systematic Reviews of Interventions (available at: http://handbook.cochrane.org/) to assess RCT quality. Bias risks for each study were assessed based on 6 factors: random sequence generation, allocation concealment, blinding (performance bias, detection bias), incomplete outcome data (attrition bias), selective reporting (reporting bias), and other bias. They were also ranked as either high risk, low risk, or unclear risk.

### 2.7. Data Synthesis and Analysis

#### 2.7.1. Direct Pairwise Meta-Analysis

We first used Review Manager version 5.3 (Cochrane Collaboration, Oxford, United Kingdom) to pool the data. Mean difference (MD) with 95% confidence intervals (CI) was used to analyze continuous data. The standardized mean difference (SMD) was used when the measurement scales between the different studies could not be corrected by conversion into a single unit of measurement, suggesting an error in the unit reported or a difference in the methods used for measurements [27]. We used the data of posttreatment for analysis. For studies that collected data on a series of time points, only the data from the first time point (immediately after intervention) was included in meta-analysis. Review Manager was also used to perform standard pairwise meta-analysis. We calculated an *I*-square (*I*^2^) test to assess heterogeneity among RCTs [28]. When *I*^2^ > 50%, they were analyzed using a random-effects model; otherwise, a fixed-effects model was used. Subgroup analyses were conducted according to the type of nonhormonal therapies and the treatment given to the control group. Predefined subgroup analysis and sensitivity analysis were performed regardless of the value of *I*^2^. We generated forest plots to illustrate the relative strength of curative effects. *P* value less than 0.05 was deemed statistically significant.

#### 2.7.2. Network Meta-Analysis

We conducted a network meta-analysis (NMA) to estimate the effect for each class and for each individual intervention using Markov chain Monte Carlo methods implemented in WinBUGS (version 1.4.3, MRC Biostatistics Unit, Cambridge, UK) [29]. Two chains with different initial values were run simultaneously to assess convergence using Brooks–Gelman–Rubin diagnostic plots. We utilized the Markov chains for 50,000 simultaneous iterations after the first 5,000 iterations were discarded because they may have had an influence on the arbitrary values. We decided whether to use a fixed-effects or a random-effects approach based on model fit statistics and deviance information criteria (DIC) [30], as well as the amount of heterogeneity present in the pairwise meta-analyses. A model with lower DIC values was preferred, with differences of 3 or more units considered meaningful. If 2 models had similar DIC values, the simplest model (i.e., the fixed-effects model) was preferred. For the network meta-analysis, we assessed the extent to which direct and indirect evidence were consistent, both qualitatively and statistically [30].

## 3. Results

### 3.1. Study Selection

**Figure 1:**
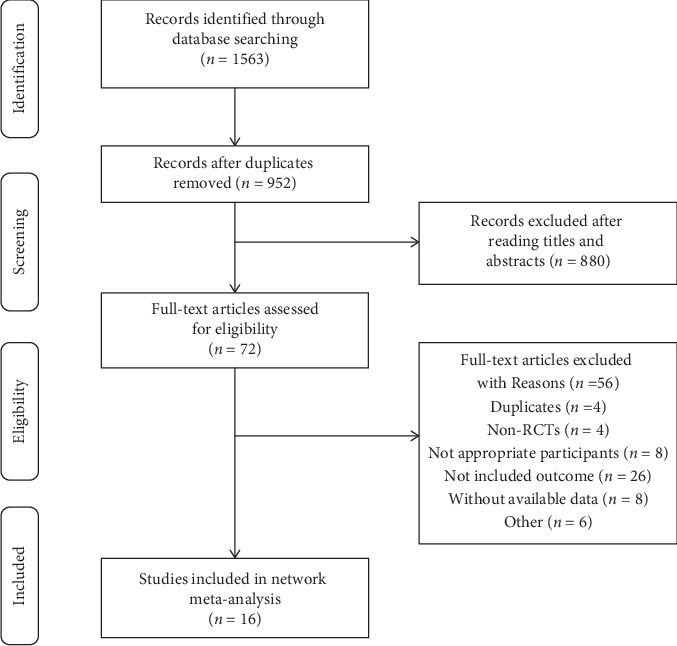
PRISMA flowchart.

As shown in Figure 1, a total of 1,563 records were initially identified from all the databases. After removing duplicate publications, 952 studies were left. 879 records remained after scanning the titles and abstracts. 73 of the full-text articles were reviewed for eligibility. 16 trials [10–16, 31–39] were included in our final NMA.

### 3.2. Study Characteristics

The 16 RCTs involving 2,349 participants included in the analyses were published between 2002 and 2016. Participants' ages ranged from 27 to 80 years, while the study sample size ranged from 37 [11] to 422 [33]. Among the included RCTs, there was 1 four-arm trial [14], 3 three-arm trials [33–35], and 12 two-arm trials. Of these studies, 14 [10–13, 15, 16, 32–39] reported hot flash frequency, while 9 [11, 12, 14, 16, 31, 32, 34, 35, 37] reported hot flash scores. The nonhormonal therapies (numbers) of the included studies were classified as follows: lifestyle changes: yoga (1), physical exercise (1); mind-body techniques: hypnosis (1), cognitive behavioral therapy (CBT) (2); dietary/supplements: soy beverages (1), black cohosh (1), melatonin (1), magnesium oxide (1); SSRIs/SNRIs: sertraline (1); other medications: gabapentin (1); other therapies: acupuncture (6). Nonhormonal therapies details, retention time, frequency, and duration of therapy are shown in Table 1.

### 3.3. Risk of Bias in Included Studies

The methodological quality of the included studies was generally poor. Most studies (12, 75%) used either a random number table or computer software for sequence generation and had been assessed as low risk of bias. Eight studies were assessed as unclear risk of bias for sequence generation and allocation concealment because there had been no clear description. Participants in seven studies were unlikely to have been blinded, due to the nature of some of the interventions and comparators, and personnel had not been blinded to group allocation. Therefore, these studies were assessed as high risk for blinding of participants and personnel. Nine studies reported that outcome assessments had been evaluated by a third party who had been unaware of the interventions; these were judged as of low risk. All studies were assessed as low risk for incomplete outcome data, as reasons for drop-out were reported, and drop-out numbers were consistent across groups. All but 1 of the studies were assessed as low risk for selective outcome reporting. The 1 exception was assessed as unclear risk because reported trial registration and published protocol cannot be identified. Uncertainty regarding the other bias was due mostly to insufficient reporting. Risk of bias assessments for all the included studies are summarized in Supplementary Material 2.

### 3.4. Pairwise Meta-Analyses

We performed meta-analyses on the different outcomes for hot flash frequency and hot flash score.

### 3.5. Hot Flash Frequency

**Figure 2:**
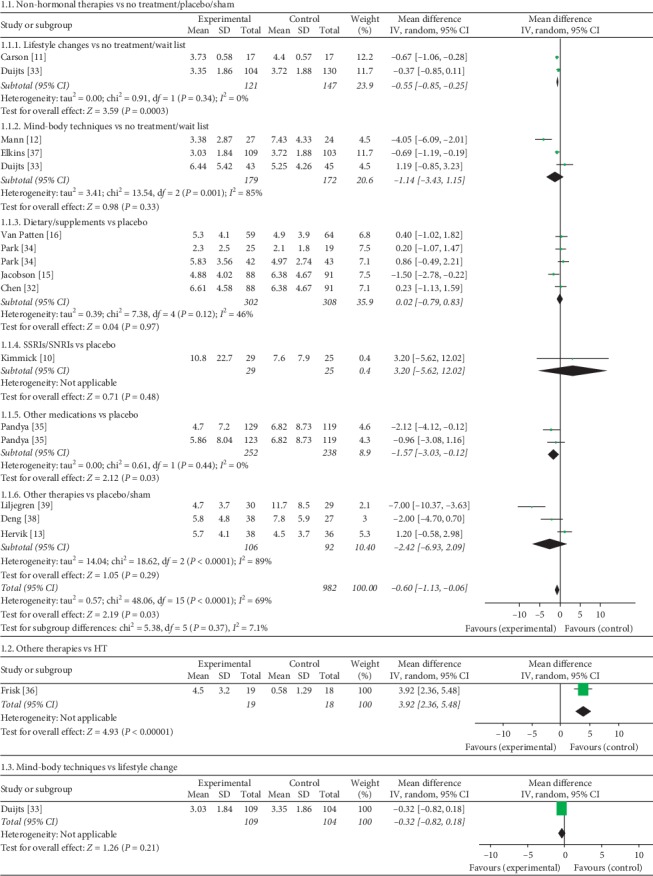
Forest plot of hot flash frequency: “any intervention that includes nonhormonal management” vs. “any intervention that does not include nonhormonal management.” CI: confidence interval; IV: inverse variance; SD: standard deviation; Std. mean difference: standardized mean difference.

Overall, the 6 nonhormonal therapies mentioned above were more efficacious in reducing hot flash frequency than no treatment/placebo/sham (SMD = −0.60, 95% CI [−1.13, −0.06]; *P*=0.03). Acupuncture, classified into other therapies, was 3.92 points less effective in reducing hot flash frequency than HT (MD = 3.92, 95% CI [2.36, 5.48], *P* < 0.00001). There was no statistically significant difference between mind-body techniques and lifestyle changes (MD = −0.32, 95% CI [−0.82, 0.18]). The mean, standard deviation (SD), and sample size of the groups are shown in Figure 2.

### 3.6. Hot Flash Score

**Figure 3:**
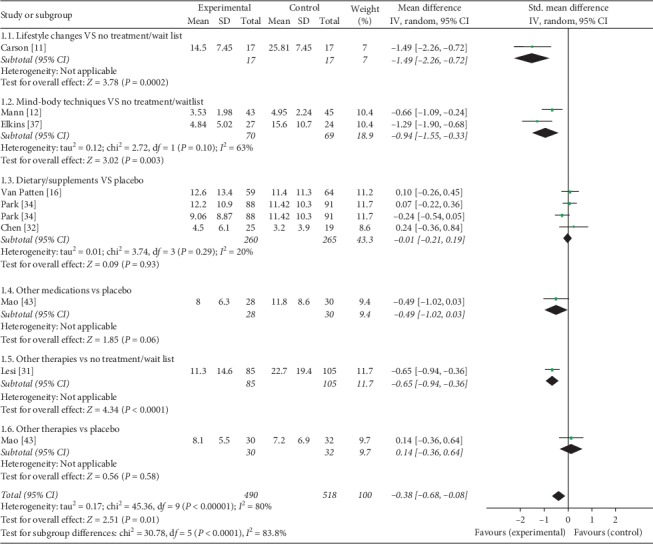
Forest plot of hot flash score: “any intervention that includes nonhormonal management” vs. “any intervention that does not include nonhormonal management.” CI: confidence interval; IV: inverse variance; SD: standard deviation; Std. mean difference: standardized mean difference.

Nonhormonal management was significantly more effective than no treatment/placebo/sham (SMD = −0.38, 95% CI [−0.68, −0.08]; *P*=0.01). This effect was particularly noticeable for lifestyle changes (SMD = −1.49, 95% CI [−2.26, −0.72]; *P*=0.00002), mind-body techniques (SMD = −0.94, 95% CI [−1.55, −0.33]; *P*=0.003), and acupuncture (SMD = −0.65, 95% CI [−0.94, −0.36]; *P* < 0.0001). There was no statistically significant difference between placebo and the following nonhormonal management treatments: dietary/supplements (SMD = −0.01, 95% CI [−0.21, 0.19]), other medications (SMD = −0.49, 95% CI [−1.02, 0.03]). Mean, standard deviation (SD) and sample size of the groups are shown in Figure 3.

### 3.7. Network Meta-Analysis

#### 3.7.1. Hot Flash Frequency

The NMA showed that nonhormonal therapies were more efficacious than no treatment/waitlist, but less effective than HT. Meanwhile, there was no statistically significant difference in hot flash frequency between any two of the treatments.

#### 3.7.2. Hot Flash Score

The results suggest that nonhormonal therapies were more effective than no treatment/waitlist, with statistically significant difference for two of the therapies: other therapies (acupuncture) (SMD = −16.05, 95% CI [−30.2, −1.99]) and lifestyle changes (SMD = −11.28, 95% CI [−20.09, −2.52]). Placebo/sham could decrease hot flash score by 12.27 points more than no treatment/waitlist (SMD = 12.27, 95% CI [−11.99, 4.43]).

**Figure 4:**
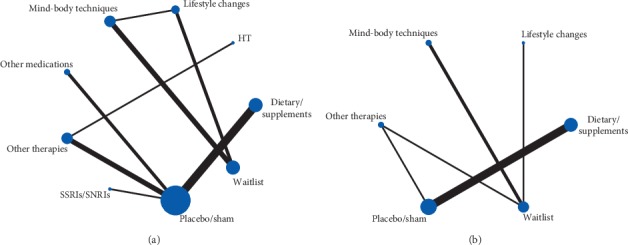
Network plot: (a) hot flash frequency; (b) hot flash score.

The results of the network meta-analysis are shown in Table 2 and the network diagram of the interventions is shown in Figure 4.

### 3.8. Ranking

#### 3.8.1. Hot Flash Frequency

The rankings of the various nonhormonal management interventions are displayed in Table 3. For reducing hot flash frequency of breast cancer survivors, other therapies (acupuncture) were ranked the optimal method, followed by other medications (gabapentin). The remainders were ranked as follows: dietary/supplements, mind-body techniques, lifestyle changes, and SSRIs/SNRIs, which were ranked after placebo/sham.

The ranking of the interventions is shown in Table 3.

#### 3.8.2. Hot Flash Score

The rankings of the various nonhormonal treatment methods are displayed in Table 3. In terms of hot flash score, acupuncture (other therapies) was also ranked the optimal therapy, followed by lifestyle changes, other medications, dietary/supplements, and mind-body techniques. The last four methods were also ranked after placebo/sham.

### 3.9. Inconsistency Assessment

#### 3.9.1. Hot Flash Frequency

For the inconsistency test outcome of hot flash frequency, 95% CI of all loops included 0, which showed that no significant inconsistency was found. However, other loops (lifestyle changes, mind-body techniques, no treatment/waitlist) were found to have statistical inconsistency between direct and indirect comparisons. The results are shown in Supplementary Material 3.

#### 3.9.2. Hot Flash Score

A *Z*-test illustrates the inconsistency of the NMA. 95% CI of all loops included 0 for their hot flash scores, which indicated that no significant inconsistency had been found. Also, there was no loop statistical inconsistency between direct and indirect comparisons.

### 3.10. Safety

Nine included studies [11, 13, 33–37, 39] reported that no adverse events had been found in either the intervention group or the control group. 8 RCTs [10, 12, 14–16, 31, 32, 38] reported adverse events. 1 study [15] reported 3 serious adverse events: hysterectomy, breast recurrence, and appendectomy, but these were not related to the intervention medicine black cohosh. 1 study [12] indicated that none of the adverse events were related to CBT. The 2 RCTs [16, 32] using dairy interventions (soy beverages, melatonin) showed more digestive system effects than the placebo. 1 study [10] using sertraline (SSRIs) showed more side effects such as nausea and diarrhea than the placebo did. 3 RCTs [14, 31, 38] reported adverse events related to acupuncture such as muscle pain and bruising.

### 3.11. Small-Sample Effect Detection

A comparison-correction funnel plot of hot flash frequency was constructed for the 15 included studies (involving 9 interventions) as shown in Supplementary Material 4. The funnel plot was asymmetrical, and 5 studies fell outside its boundaries, indicating that there might have been a small-sample effect in this study.

## 4. Discussion

### 4.1. Summary of Evidence

In this network meta-analysis, we assessed the clinical effectiveness and safety of currently available nonhormonal hot flash therapies for breast cancer survivors. We found that acupuncture was widely used in the included studies and was also the highest-ranking nonhormonal therapy for reducing hot flash frequency and score. Experimental data suggests that acupuncture reduces hot flashes. It also seems to increase central endorphin activity which may make thermoregulation more stable and in turn decrease vasomotor symptoms [40, 41]. Most clinical evidence indicates that acupuncture reduces hot flashes more than sham control groups [42, 43]. This is consistent with our results. Furthermore, our network meta-analysis also showed that placebo ranked higher than treatments such as lifestyle changes, dietary/supplements, and mind-body techniques. Some clinical evidence was consistent with these results [16, 32]. Therefore, we think that there is a high placebo response rate during hot flash treatment [44, 45]. SSRIs/SNRIs ranked lower than all other treatments except no treatment/waitlist. As antidepressants, SSRIs/SNRIs may not be fit for hot flash treatment [46]. The safety analysis showed that there had been a few related adverse events during acupuncture treatment and that drug related adverse reactions could have also occurred in studies using drug interventions. Overall, the confidence level for this review was limited. Thus, no confirmative recommendations of nonhormonal therapies for reducing hot flashes in patients with breast cancer can be made at present. Acupuncture may be effective, but more research is needed to determine whether it should be recommended.

### 4.2. Strengths and Limitations

This research has several strengths. First, this is the first network meta-analysis to investigate the effects and side effects of nonhormonal hot flash therapies for breast cancer survivors. Second, an extensive and sensitive search strategy was used to retrieve as many relevant studies as possible in this systematic review. Third, we conducted a preliminary network meta-analysis for pairwise comparison between the interventions identified. We identified many treatment options, but few studies that directly compared their effectiveness. Under these circumstances, network meta-analysis can provide indirect and mixed effect estimates that allow for comparison of the effects of different interventions [47]. The results of the indirect pairwise comparison give some insight into the potential effectiveness of each intervention, although our network meta-analysis had limited value in this study because of the small quantity of randomized controlled trials included and the potential oversight of relevant interventions.

This research also has several limitations. First, this systematic review was limited by the small number of randomized clinical trials comparing hot flash treatments. Few pairs of treatments had been compared in more than one trial. Therefore, we classified specific treatments according to the statement [21] and then did analysis, which may have affected the results and increased limitations for clinical guidance. Second, we failed to evaluate the safety of each therapy, due to limited data in the primary studies. Future trials should report the relative effectiveness and side-effect profiles of active treatments in order to determine the best clinical management approach. Third, 50% of studies were assessed as 'unclear' in allocation concealment. As the hot flush frequency and score are patient-reported outcomes, if there is no blinding (participants and personnel), this could cause no blinding on the outcome assessors, which implies the possibility of detection bias and performance bias. Therefore, methodological quality should be paid more attention when blinding is limited due to the nature of some interventions in future studies. In addition, despite the fairly consistent collection of hot flash frequency and score data using hot flash diaries, studies did not consistently report outcomes in comparable formats. This prohibited the pooling of estimates in meta-analysis. There was great variability in how hot flash outcomes were reported statistically, such as means and standard deviations or standard errors, means and 95% confidence intervals, and medians and ranges. Future studies should report hot flash frequency and score results using a common measure, so that their results can be compared directly.

## 5. Conclusion

This network meta-analysis compares nonhormonal management interventions for hot flashes in breast cancer survivors. Our network meta-analysis suggests that acupuncture has been the most frequently tested intervention and might be more effective in improving hot flashes for breast cancer survivors. Pronounced placebo response was also found in hot flash treatment. The evidence of safety for the nonhormonal therapies is also insufficient. Therefore, no confirmative recommendations of nonhormonal management for hot flashes in breast cancer survivors can be made at present. Further research is needed to determine the recommendations that can be made when enough clinical studies of hot flashes have been conducted.

## Figures and Tables

**Table 1:** Characteristics of the 16 included studies.

Reference	Participants	Interventions and comparisons	Outcomes measures
Van Patten et al. [16]	123 participants (38 on tamoxifen) (i) Breast cancer stages: I–III (ii) Menopausal (≥12 months of amenorrhea) (iii) Not using HRT for ≥4 months (iv) Hot flashes with a score (frequency × intensity) of ≥10/wk	12-week study 2 groups: (i) Soy beverage (containing 90 mg of isoflavones), 500 ml daily (ii) Placebo (a rice beverage)	① + ②

Jacobson et al. [15]	89 participants (59 on tamoxifen) (i) Age ≥18 (ii) Completing primary therapy—before entering the trial ≥2 months	60-day study 2 groups: (i) Black cohosh, one tablet twice daily (ii) Placebo	①

Lesi et al. [31]	190 participants (170 on HT) (i) Age 18 to 65 years (ii) Spontaneous or induced (iii) Amenorrhea ≥6 months (iv) Hot flashes mean number ≥6	12-week study 2 groups: (i) Acupuncture once per week (ii) Waitlist	②

Hervik et al. [13]	95 participants (i) Tamoxifen used ≥3 months (ii) No menstruation ≥3 months	10-week study 2 groups: (i) Acupuncture (ii) Sham acupuncture Both twice weekly for the first 5 weeks, once a week for the following 5 weeks	①

Frisk et al. [36]	45 participants (i) Breast cancer stages: in situ, T1 and T2 tumors with four or fewer lymph nodes positive for metastasis, T3 tumors without metastasis	12-week study 2 groups: (i) Electroacupuncture (EA) twice a week for the first 2 weeks, and once a week for 10 weeks (ii) Hormone therapy (HT) estrogen/progestagen combination or continuous combined estrogen/progestagen	①

Mao et al. [14]	120 participants (i) Breast cancer stages: I–III (ii) Hot flashes each day ≥2 (iii) Hot flashes presented ≥1 month (iv) Nonhormonal contraceptives	8-week study 4 groups: (i) Electroacupuncture (EA) (ii) Sham acupuncture Both twice per week for 2 weeks, then once per week for 6 more weeks (iii) Gabapentin (GP) (iv) Placebo Both one pill (300 mg) at bedtime for 3 days, then twice per day for 3 days, and then three times per day for the remaining 50 days	②

Elkins et al. [37]	51 participants (i) Age ≥18 (ii) Hot flashes per week ≥14 (iii) Hot flashes presented ≥1 month	5-week study 2 groups: (i) Hypnosis intervention five weekly sessions, each to last approximately 50 minutes (ii) No treatment	① + ②

Deng et al. [38]	72 participants (i) Karnofsky performance score ≥60 (ii) Hot flashes each day ≥3	4-week study 2 groups: (i) Acupuncture (ii) Sham acupuncture Both twice per week	①

Liljegren et al. [39]	74 participants (i) Vasomotor symptoms questionnaire score ≥5 (ii) Tamoxifen used ≥2 months	4-week study 2 groups: (i) Acupuncture (ii) Control acupuncture Both twice per week	①

Carson et al. [11]	37 participants (i) Breast cancer stages: IA–IIB (ii) Hot flashes each day, ≥2 (ii) No hormone replacement therapy and no menopausal symptom medications, currently or within prior 3 months	8-week study 2 groups: (i) Yoga, eight weekly 120-min group classes (5–10 patients per group) (ii) Waitlist	① + ②

Duijts et al. [33]	422 participants (i) Breast cancer stages: T1-4, N0-1, and M0 (ii) Age <50 y (ii) Premenopausal women (iii) Receiving adjuvant chemotherapy and/or hormonal therapy (iv) Chemotherapy completed during 4 months to 5 years (iv) Hormonal therapy ongoing	12-week study 3 groups: (i) Cognitive behavioral therapy (CBT), six weekly group sessions of 90 minutes each (ii) Physical exercise (PE) 12-week, individually tailored, home-based, self-directed exercise program of 2.5 to 3 hours per week (iii) Waiting list	①

Eleanor Mann et al. [12]	96 participants (i) Age ≥18 (ii) Completed medical treatment for breast cancer adjuvant endocrine treatment	9-week study 2 groups: (i) Cognitive behavioral therapy (CBT) (ii) Waiting list	① + ②

Chen et al. [32]	95 participants (i) Breast cancer stages: I–III (ii) Hormonal therapy at least 60 days prior to enrollment	4-months study 2 groups: (i) Melatonin 3 mg daily (ii) Placebo	① + ②

Park et al. [34]	358 participants (i) Postmenopausal women (ii) Hot flashes each week ≥14 (iii) Hot flashes presented for ≥ days	8-week study 3 groups: (i) Magnesium oxide 800 mg daily (ii) Magnesium oxide 1200 mg daily (iii) Placebo	① + ②

Kimmick et al. [10]	62 participants (i) Age ≥18 (ii) Breast cancer stages: 0–IIIB (iii) Adjuvant tamoxifen therapy (iv) Hot flashes per day ≥1	6-week study 2 groups: (i) Sertraline, 50 mg each morning (ii) Placebo	①

Pandya et al. [35]	420 participants (i) Age ≥18 (ii) Hot flashes each day ≥2 (iii) Nonsteroidal contraceptive measures (iv) Patients currently receiving chemotherapy were not eligible, although endocrine therapies were allowed	8-week study 3 groups: (i) Gabapentin 900 daily (ii) Gabapentin 300 daily (iii) Placebo	① + ②

① indicates hot flash frequency. ② indicates hot flash score.

**Table 2:** Results of network meta-analysis.

Hot flash frequency								
Lifestyle changes								
0.53 (−3.44, 4.51)	Mind-body techniques							
5.69 (−66.24, 79.85)	5.17 (−66.73, 79.47)	Dietary/supplements						
2.59 (−70.16, 76.88)	2.06 (−70.72, 76.47)	−3.1 (−13.54, 7.32)	SSRIs/SNRIs					
7.79 (−64.32, 82.02)	7.27 (−64.95, 81.44)	2.1 (−3.92, 8.13)	5.21 (−6.22, 16.66)	Other medications				
7.82 (−64.1, 82.14)	7.3 (−64.78, 81.74)	2.13 (−1.93, 6.51)	5.24 (−5.29, 15.93)	0.03 (−6.19, 6.5)	Other therapies			
11.73 (−60.44, 86.28)	11.21 (−61.13, 85.78)	6.04 (−0.56, 12.9)	9.15 (−2.52, 21.09)	3.94 (−4.18, 12.3)	3.91 (−1.36, 9.16)	HT		
−0.64 (−4.1, 2.83)	−1.16 (−4.19, 1.81)	−6.33 (−80.56, 65.69)	−3.22 (−77.68, 69.63)	−8.43 (−82.57, 63.74)	−8.46 (−82.86, 63.46)	−12.37 (−86.86, 59.93)	No treatment	
5.67 (−66.27, 79.88)	5.15 (−66.85, 79.47)	−0.02 (−2.65, 2.61)	−2.12 (−7.56, 3.3)	−1.54 (−5.08, 2)	−2.15 (−5.55, 0.98)	−6.06 (−12.38, 0.03)	6.31 (−65.74, 80.62)	Placebo/sham

Hot flash score								

Lifestyle changes								
−7.01 (−17.45, 3.66)	Mind-body techniques							
0.81 (−14.34, 15.87)	7.82 (−5.87, 21.2)	Dietary/supplements						
4.76 (−11.83, 21.44)	11.77 (−3.64, 26.98)	3.95 (−5.19, 13.07)	Other medications					
0.08 (−12.2, 12.27)	7.09 (−3.43, 17.37)	−0.73 (−9.43, 8.13)	−4.68 (−15.97, 6.6)	Other therapies				
**−11.28 (−20.09, −2.52)** ^**∗**^	−4.28 (−10.28, 1.42)	−12.09 (−24.32, 0.08)	**−16.05 (−30.2, −1.99)** ^**∗**^	−11.37 (−19.96,−2.87)	No treatment			
0.98 (−13.66, 15.58)	7.99 (−5.03, 20.8)	0.17 (−3.8, 4.16)	−3.78 (−11.99, 4.43)	0.9 (−6.98, 8.71)	**12.27 (0.78, 23.76)** ^**∗**^	Placebo/sham		

^*∗*^Statistically significant difference between two therapies.

**Table 3:** Ranking of interventions and comparisons.

Interventions	Hot flash frequency		Hot flash score	
	WinBUGS results	Ranking	WinBUGS results	Ranking
Lifestyle changes	0.150	7	0.0071	3
Mind-body techniques	0.071	6	0.064	6
Dietary/supplements	0.066	5	0.018	5
SSRIs/SNRIs	0.327	9	—	—
Other medications	0.029	3	0.009	4
Other therapies (acupuncture)	0.007	2	0.003	1
HT	0.003	1	—	—
Placebo/sham	0.047	4	0.006	2
No treatment/waitlist	0.300	8	0.894	7

## Supplementary Materials

Supplementary Materials Supplementary Material 1: search strategies for RCTs on nonhormonal management for breast cancer survivors in PubMed. Supplementary Material 2: risk of bias graph. Supplementary Material 3: the figure of inconsistency assessment for the outcome of hot flash frequency. Supplementary Material 4: the funnel plot of comparison, “any nonhormonal intervention versus no nonhormonal intervention,” for the outcome of hot flash frequency.
