# Supplementary material for: Nonhormonal Hot Flash Management for Breast Cancer Survivors: A Systematic Review and Network Meta-Analysis
Source: Evid Based Complement Alternat Med. 2020 Apr 28;2020:4243175. doi: 10.1155/2020/4243175 (PMC7204350; doi:10.1155/2020/4243175)
Supplement: Supplementary Materials — Supplementary Material 1: search strategies for RCTs on nonhormonal management for breast cancer survivors in PubMed. Supplementary Material 2: risk of bias graph. Supplementary Material 3: the figure of inconsistency assessment for the outcome of hot flash frequency. Supplementary Material 4: the funnel plot of comparison, “any nonhormonal intervention versus no nonhormonal intervention,” for the outcome of hot flash frequency. [file 4243175.f1.pdf]

Additional file 1: Search string in PubMed

1. “Breast Neoplasms”[Mesh] NOT “Breast Neoplasms, Male”[Mesh]
2. “Breast cancer” OR “Breast Neoplasms”
3. OR/1-2
4. “Hot Flashes”[Mesh]
5. “hot flashes” OR “hot flush” OR “vasomotor symptoms” OR “night sweats” OR “menopausal symptoms”
6. OR/4-5
7. “Clinical Trial”[Publication Type]
8. “Phase I Clinical Trial” OR “Phase II Clinical Trial” OR “Phase III Clinical Trial” OR “Phase IV Clinical Trial” OR “Controlled Clinical Trial” OR “Multicenter Study” OR “Randomized Controlled Trial” OR “Pragmatic Clinical Trial”
9. OR/7-8
10. “Serotonin Uptake Inhibitors”[Mesh] OR “Neurotransmitter Uptake Inhibitors”[Mesh] OR “Citalopram”[Mesh] OR “Fluvoxamine”[Mesh] OR “Paroxetine”[Mesh] OR “Fluoxetine”[Mesh] OR “Sertraline”[Mesh] OR “Duloxetine”[Supplementary Concept] OR “Venlafaxine”[Supplementary Concept]
11. “Venlafaxine” [Supplementary Concept]
12. “Serotonin Uptake Inhibitors” OR “Celexa” OR “citalopram” OR “citalopram hydrobromide” OR “Lexapro” OR “escitalopram oxalate” OR “fluvoxamine” OR “fluvoxamine maleate” OR “Paxil” OR “paroxetine hydrochloride” OR “Prozac” OR “fluoxetine hydrochloride” OR “Zoloft” OR “sertraline” OR “sertraline hydrochloride” OR “duloxetine” OR “Cymbalta” OR “Effexor XR” OR “venlafaxine” OR “Pristiq” OR “desvenlafaxine” OR “desvenlafaxine succinate”
13. “Gabapentin” [Supplementary Concept] OR “Pregabalin” [Supplementary Concept]
14. “gapapentin” OR “pregabalin” OR “neurontin” OR “gralise”
15. “Clonidine” [Mesh]
16. “clonidine” OR “catapres” OR “catapres-TTS”
17. “Homeopathy” [Mesh] OR “Vitamin E” [Mesh] OR “Cimicifuga” [Mesh] OR “Klimadynon” [Supplementary Concept] OR “Cimicifuga extract BNO 1055” [Supplementary Concept]
18. “Non-hormonal treatments” OR “homeopathy” OR “Vitamin E” OR “black cohosh” OR “cimicifuga”
19. “Yoga” [Mesh] OR “Meditation” [Mesh] OR “Exercise” [Mesh] OR “Relaxation Therapy” [Mesh] OR “Breathing Exercises” [Mesh] OR “Resistance Training” [Mesh]
20. “yoga” OR “meditation” OR “exercise” OR “relaxation therapy” OR “breathing exercises” OR “resistance training”
21. “Behavior Therapy”[Mesh] OR “Cognitive Therapy”[Mesh] OR “Mindfulness”[Mesh] OR “Counseling”[Mesh]
22. “Soybeans”[Mesh] OR “Isoflavones”[Mesh] OR “Phytoestrogens”[Mesh]
23. “soybeans” OR “soybean oil” OR “soy proteins” OR “soy foods” OR “soy milk” OR “soy products” OR “soy” OR “isoflavones” OR “phytoestrogens”
24. “behavior therapy” OR “behavior modification” OR “cognitive therapy” OR “cognitive behavior therapy” OR “mindfulness” OR “counseling”
25. “Flax”[Mesh] OR “Linseed Oil”[Mesh]
26. “flax” OR “flaxseed” OR “flaxseed oil” OR “linum” OR “linseed oil”
27. “Physical Therapy Modalities”[Mesh] OR “physical therapy”
28. “Acupuncture Therapy” [Mesh] or “Acupuncture” [Mesh]
29. “acupuncture” or “acupuncture therapy”
30. “Auriculotherapy”[Title/Abstract] OR “ear acupressure”[Title/Abstract]
31. “Moxibustion” [Title/Abstract] OR “moxa”[Title/Abstract]
32. “Tuina” [Title/Abstract] OR “Chinese massage”[Title/Abstract])
33. “Traditional Chinese Medicine” [Title/Abstract] OR “Chinese Medicine”[Title/Abstract] OR “Chinese Traditional Medicine”[Title/Abstract] OR “Chinese Drugs”[Title/Abstract] OR “Herbs” [Title/Abstract] OR “Chinese Medicine Herb” [Title/Abstract] OR “Herbal Medicine” [Title/Abstract])
34. OR/10-33
35. 3 AND 6 AND 9 AND 34

Additional file 2: Risk of bias graph

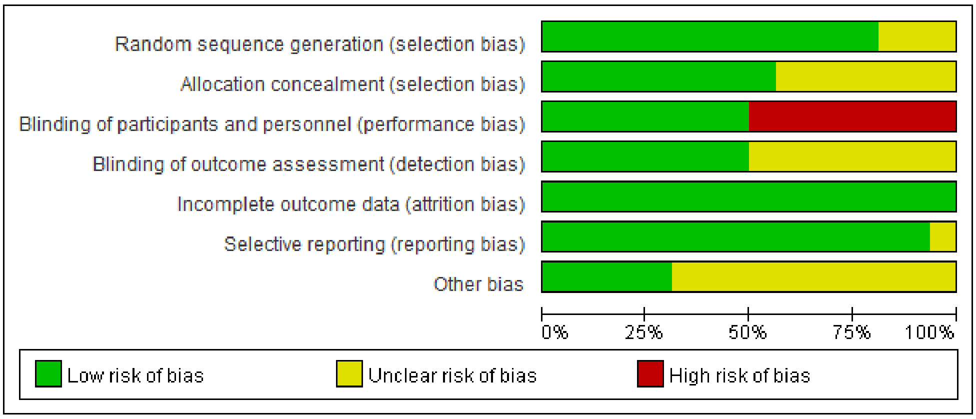

Risk of bias graph: review authors' judgements about each risk of bias item presented as percentages across all included studies

Additional file 3: Inconsistency assessment of Hot flash frequency

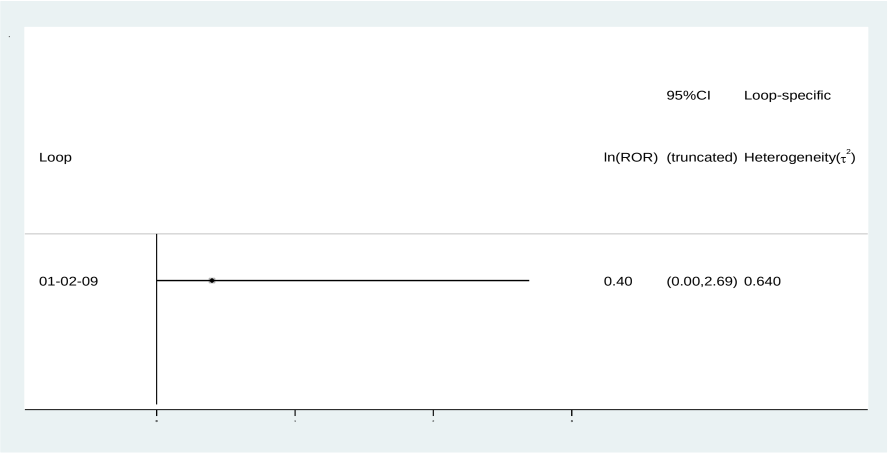

Note：

01：Lifestyle changes；02：Mind-body techniques； 09：No treatment/Waitlist；

Additional file 4: Funnel plot of hot flash frequency

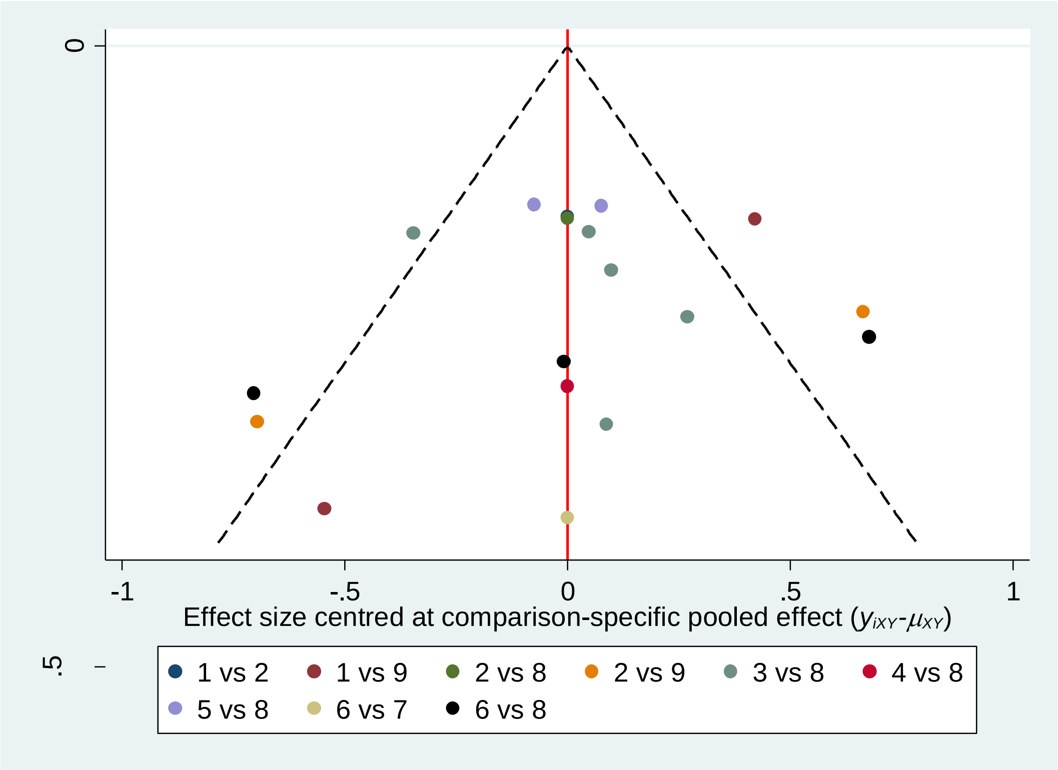

Funnel plot of comparison "Any non-hormonal intervention versus no non-hormonal intervention", outcome: Hot flash frequency

Note：

1：Lifestyle changes；2：Mind-body techniques；3：Dietary/supplements

4：SSRIs/SNRIs；   5：Other medications；   6：Other therapies (Acupuncture)

7：HT   8：Placebo/Sham；   9：No treatment/Waitlist
